# Women, Exercise, and Eating Disorder Recovery: The Normal and the Pathological

**DOI:** 10.1177/1049732321992042

**Published:** 2021-02-16

**Authors:** Hester Hockin-Boyers, Megan Warin

**Affiliations:** 1 University of Durham, Durham, United Kingdom; 2 University of Adelaide, Adelaide, South Australia, Australia

**Keywords:** eating disorder recovery, Georges Canguilhem, exercise, weightlifting, yoga, gender, normal, pathological, longitundinal qualitative interview, United Kingdom

## Abstract

The appropriate form, regularity, and intensity of exercise for individuals recovering from eating disorders is not agreed upon among health care professionals or researchers. When exercise is permitted, it is that which is mindful, embodied, and non-competitive that is considered normative. Using Canguilhem’s concepts of “the normal and the pathological” as a theoretical frame, we examine the gendered assumptions that shape medical understandings of “healthy” and “dysfunctional” exercise in the context of recovery. The data set for this article comes from longitudinal semi-structured interviews with 19 women in the United Kingdom who engaged in weightlifting during their eating disorder recovery. We argue that women in recovery navigate multiple and conflicting value systems regarding exercise. Faced with aspects of exercise that are pathologized within the eating disorder literature (such as structure/routine, body transformations, and affect regulation), women re-inscribe positive value to these experiences, thus establishing exercise practices that serve them.

## Introduction

In 2018, Hockin-Boyers attended an international conference on body image to seek feedback on her ongoing research project exploring women’s use of weightlifting as a strategy for recovery from eating disorders (EDs). The conference spanned 3 days and during this time, a great number of researchers spoke to the fraught relationship between exercise practices and ED pathology. Interestingly, in paper presentations, weightlifting and some associated practices (such as drinking protein shakes) were presented as possible indicators of ED symptomology. Moreover, when speaking with other delegates, Hockin-Boyers encountered some resistance to the notion that weightlifting could be health promoting for women—in recovery or otherwise. Underpinning this assertion was the supposition that certain forms of exercise (such as those that are structured or involve a degree of self-regulation) are problematic for people with EDs. However, not all forms of exercise were perceived in this way. In one of the keynote presentations, yoga was presented as a possible tool for recovery, a claim that those in attendance appeared generally supportive of. In this respect, exercising in ways that were non-competitive, fostered mind–body connections, were highly gendered, and were somewhat spiritually based, emerged as the “gold standard” when it came to engaging with healthy forms of movement during recovery and beyond. From attending this conference, it became clear to Hockin-Boyers that within this field, there exists an implicit understanding of what constitutes “normal” and “pathological” exercise in the context of ED recovery.

By drawing on Canguilhem’s (1943/1966) scholarship within the philosophy of medical sciences on the “normal and pathological,” we critically examine normative understandings of exercise during ED recovery. The data set from this article comes from research on women’s use of weightlifting as a vehicle for recovery from their ED. A total of 19 women were recruited, using both online and offline sampling methods, to take part in longitudinal semi-structured interviews—three with each participant over the course of 8 months. The purpose of the project was to understand the role of weightlifting in women’s experiences of recovery. The outcomes of this research, we argue, are particularly relevant to scholarship on clinical practice and ED recovery for a number of reasons. First, the women in this study have lived experience of both what clinicians might refer to as “normal” and “dysfunctional” exercise, making their insights on this topic particularly significant and novel. Second, as the participants in this sample have taken up weightlifting of their own accord, this article acts as an exploration of the kind of exercise women themselves gravitate toward as they transition out of this difficult phase in their lives. In this regard, this article is guided by two central questions.

**Research Question 1:** For the women in this study, what constitutes “normal” and “pathological” exercise and how is this navigated during ED recovery?
**Research Question 2:** For the women in this study, what is it about weightlifting that makes this sport a desirable form of exercise during ED recovery?

In what follows, we begin by detailing how exercise is constructed as “dysfunctional” in ED diagnosis and during recovery, as well as the more limited work that has been conducted to conceptualize “healthy” exercise in this context. We then discuss the insights Canguilhem’s (1943/1966) theoretical framework can bring to bear on this topic, before outlining the study and methods. The findings are divided into three key themes pertaining to exercise during ED recovery: (a) structure and managing routines, (b) what the body can do, and (c) regulating affect. In critically assessing weightlifting through Canguilhem’s (1943/1966) “normal and pathological” framework, we trace the ways in which participants negotiated differing (and often conflicting) norms around exercise. In particular, we draw out tensions between clinical understandings of dysfunctional exercise practices and gendered cultural values that normalize disciplining the body through sport, attending to how women in recovery subjectively navigate this conflict in their everyday lives. Moreover, as EDs (and their treatment) are highly gendered (Eli, 2018; Lester, 2019; Moulding, 2006; Musolino et al., 2020), throughout we interrogate the role of gender in clinical conceptions of the “recovered” feminine subject and how this translates into understandings of “healthy” movement. We end by discussing the implications of broadened options and understandings of exercise in ED recovery for future scholarship and practice.

## Exercise: The Normal and the Pathological

### “Dysfunctional” Exercise

In academic scholarship, what constitutes pathology with respect to eating and exercise behavior has traditionally been defined by clinical psychiatrists and psychologists. The *Diagnostic and Statistical Manual of Mental Disorders* (5th ed.; *DSM*-5; American Psychiatric Association, 2013), written and compiled by the American Psychiatric Association, is one key site through which researchers and practitioners keep abreast of developments in psychiatry and current understandings of mental ill-health. Although dysfunctional exercise is not included in the *DSM*-5 as a disorder in and of itself, “excessive exercise” has long been listed as a symptom of anorexia and bulimia nervosa (American Psychiatric Association, 2013).

In the ED literature more broadly, problematic relationships to exercise have multiple names and definitions. These include; “activity anorexia” (Epling et al., 1983), “exercise anorexia” (Touyz et al., 1987), “obligatory exercise” (Davis et al., 1993), “exercise addiction” (Freimuth et al., 2011), “exercise dependence” (Bratland-Sanda et al., 2011), “exercise abuse” (Calogero & Pedrotty, 2004), “excessive exercise” (Mond et al., 2006), and “anorexia athletica” (Sudi et al., 2004). For the sake of simplicity, we follow Calogero and Pedrotty-Stump (2010), who have written extensively on exercise during ED recovery, by using “dysfunctional exercise” as an umbrella term to capture these multiple labels and definitions. It is noteworthy to mention that we are mobilizing the language of “dysfunction” for the purpose of framing our findings within Canguilhem’s concepts of the normal and the pathological and are critical of the limitations held by this binary categorization.

As noted in the introduction, there are troubling links between dysfunctional exercise and EDs that justifiably arouse caution in both health care professionals and researchers alike. The clinical literature states that it is common for problematic relationships toward exercise to co-occur with EDs (Dalle Grave et al., 2008; Freimuth et al., 2011). There is also evidence to suggest that dysfunctional exercise can precede the onset of EDs and is one of the last symptoms to subside (Calogero & Pedrotty, 2004). Moreover, within a clinical context, high levels of dysfunctional exercise at discharge is said to predict a quicker relapse and a chronic outcome among patients with anorexia nervosa (Mond et al., 2006; Strober et al., 1997). In this respect, dysfunctional exercise and ED pathology are closely linked and feed into one another in ways that demand careful attention.

When defining dysfunctional exercise, scholarship has moved away from metrics such as volume, intensity, and regularity of exercise due to the consequent overlap between dysfunctional behaviors and the lifestyles of elite athletes (Freimuth et al., 2011). Instead, the literature typically defines dysfunctional exercise as a set of attitudes, motivations, and beliefs (Davies et al., 2008). Although definitions vary, commonly cited features of dysfunctional exercise include (yet are not limited to) exercising for appearance reasons, affect regulation, identity maintenance, self-harm, permission to eat, when physical well-being and/or safety are compromised, and to keep a rigid routine (Calogero & Pedrotty, 2004).

In addition to psychiatric understandings of dysfunctional exercise, there is a wealth of literature from a feminist sociological perspective which is highly critical of the cultural valuing of exercise as a disciplinary technique (Bartky, 1988; Marzano-Parisoli, 2001). Drawing on Foucault’s (1976/1977) theories of governmentality and technologies of self, this work calls into question the role of exercise in contemporary society, positioning it as a tool used to regulate and control bodies through internalized norms and behaviors. Thus, women engage in a process of self-subjectification, viewing their body as something in need of continuous work, improvement, and management. In the United Kingdom and much of the West, this system of governance has arguably intensified, marked by the emergence of neoliberal public health agendas which encourage individuals to take up responsibility for their health, despite living within conditions which are not health promoting (Musolino et al., 2020; Pirie, 2016). Crucially, it is within this climate of self-regulation and normalized bodily discipline that women must undertake their recovery from EDs, which makes exercise in recovery a difficult terrain to navigate (Gill & Orgad, 2018; Rose, 2014).

### When Is Exercise “Healthy”?

While there are multiple definitions of dysfunctional exercise, there is much less writing on what might constitute *healthy* exercise in the context of ED recovery and beyond (Davies et al., 2008). This lack of theorizing the healthful body has been noted by other scholars, for example, Monaghan (2001) writes, “even among those medical sociologists who stress the importance of theorizing the body, primary emphasis is given to sickness, disability and death as opposed to vibrant physicality and associated embodied pleasures” (p. 331). This bias toward research on poor health is justified given the underpinning inequalities associated with illness and the relative privileged position occupied by those who enjoy good health. However, the absence of such definitions in the context of ED research raises questions as to whether clinicians believe such a thing to be achievable for this population.

Researchers in this field have noted that the task of differentiating “healthy” from “dysfunctional” exercise is challenging. As Freimuth and colleagues (2011) note,

healthy exercise can share attributes of an addiction. There can be tolerance in which a person runs farther or lifts more weight before feeling gratified that the workout was worthwhile. Normal exercise does not preclude creating negative consequences in the form of physical injury or time taken away from other important activities. (pp. 4072–4073)

Moreover, work by Musolino and colleagues (2020) on the experiences of women with severe and enduring anorexia shows that accepted norms within health and fitness culture (what they refer to as the “healthism *habitus*”; Musolino et al., 2015) can easily be categorized by clinicians as pathological practices. In their study, they describe how a participant with a long-standing ED diagnosis found discussions about food and exercise with her personal trainer at the gym to be normalized in a discourse of fitness, control, and health. Perceptively, this participant noted that when she described her same practices to health professionals, she was pathologized as “sick” (Musolino et al., 2020).

This ambiguity with respect to the dividing lines between normal and pathological exercise is reflected in the lack of clear guidance in health care settings. A 2008 survey of 43 ED treatment units in the United Kingdom revealed that 53% of units have written documentation on the management of physical activity and only 9% have a written definition of what constitutes healthy exercise (Davies et al., 2008). The challenge practitioners face in defining healthy exercise speaks to Canguilhem’s (1943/1966) critique of the ways in which “the normal and the pathological” have been arranged into a binary where one is pitched against the other; where normal is equated with healthy and dysfunction with disease.

Understanding what constitutes positive and supportive exercise is not easily identified within this literature. However, it is possible to glean some sense of what is considered to be healthy exercise from the small number of exercise programs that have been implemented and evaluated in ED treatment settings in the United States and in Europe. In most cases, exercise in these programs tends to be gentle, non-competitive, and often taking place outdoors. For example, walking, yoga, and Pilates are regularly offered (Danielsen et al., 2018; Davies et al., 2008; Hechler et al., 2005). These activities are sometimes also accompanied by psychoeducational sessions about exercise, as well as body-oriented therapy (administered by physiotherapist) involving relaxation exercises, body awareness movements, and massages (Danielsen et al., 2018).

As was highlighted in the introduction to this article, yoga, which is framed by researchers and practitioners as having therapeutic benefits for a number of psycho-somatic issues (Hoyez, 2007), is one of the only forms of exercise that has been explicitly advocated for as both potential treatment of and prevention from EDs (Calogero et al., 2010; Douglass, 2010; McIver et al., 2009; Neumark-Sztainer, 2014; Piran & Neumark-Sztainer, 2020). Writing on the benefits that yoga might offer this population, Calogero and colleagues (2010) contend,

yoga is a particularly powerful tool for cultivating mindful exercise. The aim of yoga is to fully experience the present moment, encouraging attunement to internal sensations versus external stimuli . . . Thus, yoga may help in ED treatment and recovery because it teaches specific tools to facilitate healing. (p. 435)

It is noteworthy to add that, in addition to EDs, yoga is increasingly advocated for alongside a host of other practices (such as “mindfulness”) as a form of self-help with a range of mental health issues. Many scholars have critiqued such a neoliberal approach to health care, for placing the onus on individuals to regulate their own well-being in spite of the challenging circumstances they have found themselves in (Brijnath & Antoniades, 2016; Rose, 2014).

Despite the persistent emphasis on yoga as a tool for recovery within the ED literature, emerging evidence on the efficacy of this activity for this population remains inconclusive (Balasubramaniam et al., 2013). Moreover, some researchers have highlighted that the inclusion of mindful yoga, which teaches listening and responding to the body, in treatment settings juxtaposes with the otherwise self-regulatory nature (e.g., the monitoring of bathroom breaks and physical activity) of the environment (Douglass, 2010). Thus, yoga presents women in treatment with conflicting messages about how to relate to their bodies.

Theorizing healthy exercise for women in recovery as that which is mindful and embodied offers a highly gendered understanding of women’s well-being. As Hardes (2018) suggests in her analysis of changing medical and historical accounts of women, madness and exercise, physical activity for women’s mental health has shifted from a cause and an abstinence paradigm, to now a cure and “prescription for mental health” (p. 181). The choice of exercise for women however,

continues to be deeply normalised and embedded with gendered assumptions. Mindful forms of exercise, for example, are increasingly promoted for women’s depressive states . . . While scholars have been quick to identify the gendered nature of fitness practices like aerobics that perpetuate dominant female bodily norms (toned, thin, beautiful), less attention has been paid to the normative dimension inherent in practices that present themselves as specifically useful for women’s “emotional” ailments. (p. 191)

In ED spaces, yoga emerges as a normalized, healthy exercise, premised on gendered constructions of femininity, sociality, pleasure, and an absence of competition (Strings et al., 2019). Other activities deemed overly masculine (such as those rooted in competitive sport, like weightlifting) sit in stark contrast to the feminized exercise recommended for ED recovery (Brace-Govan, 2004). Clearly, the links between women, exercise, and mental health continue to be “profoundly shaped by wider social agendas and views on women’s bodies and minds” (Hardes, 2018, p. 191). Furthermore, the assertion that exercise is feminized within the context of ED treatment aligns with the multiple works by sociologists and anthropologists on the highly gendered nature of treatment settings (Lester, 2007; Moulding, 2006). As Lester (2007) notes, “different eating disorder treatments configure selective cultural proscriptions about agency, sexuality, desire, and femininity within the ideological and practical commitments of each setting” (p. 371).

### Canguilhem and the Differing Values of Exercise

The discourses that have been constructed around exercise in EDs—of dysfunctional or healthy—resonate with the distinction built into the binary framework of the normal and the pathological. In his book of the same name (*The Normal and the Pathological* [1943/1966]), Canguilhem argues that things should not be designated as normal or pathological simply because they are objectively so. Pitching his arguments against the positivist philosophy of Auguste Comte (1798–1857) and Claude Bernard (1813–1878), Canguilhem argued that norms should not be solely defined by objective, scientific “methods for quantifying physiological concepts” (Canguilhem, 1943/1991, p. 75). He discusses at length how Bernard drew on physiological studies on diabetes to demonstrate the science of norms:

since having glucose in the blood is a normal state, then the symptom of having excess glucose in the urine (glycosuria) implies a continuity between the normal and the pathological (Bernard, 1877). All that is different is the intensity or quantity. (Cited in Sholl, 2015, p. 406)

In Bernard’s formulation, the normal is the “ideal organism” typically studied in a laboratory and whenever a blood sugar level (or some other quantitative measure) deviates from the normal state, then it is classified as pathology. For Bernard, this was a positive fact.

Canguilhem rejected the idea that pathology is a variation of the normal state on both medical and philosophical grounds. For the purposes of our argument, we focus on his relevant points about values, lived realities, and environments. First, Canguilhem rejected the idea that norms are facts. Instead, he argued that norms are values and that different values can be attached to different states. In appreciating lived realities over facts, Canguilhem suggested that new norms are constantly created, and people create and can live with a multiplicity of norms (Mol, 1998). This leads to one of Canguilhem’s main points of disagreement with Bernard, that the interaction of people (organisms) with their environment makes norms and pathology dynamic, adaptable, and diverse, meaning they can be simultaneously valued as good, bad, or neither.

Taking Canguilhem’s foundational critique of the normal and pathological (which underpinned his student’s later work in *The Birth of The Clinic* (Foucault, 1963/1976), we can see how discourses of dysfunctional and normal have been constructed in the clinical literature concerning exercise and EDs. The value attached to forms of “excessive” exercise relate not only to clinical classifications but also to gendered norms of masculinity and femininity, with weightlifting constructed as negative and dysfunctional for women, and yoga accepted as normal and non-pathological. The key tension described at the beginning of this article captures the normalizing judgments that can arise when differing values are placed on the same activity—Weightlifting can be valued as either a health-enhancing activity or a sign of illness, as normative or pathological. Following Canguilhem, we argue that this binary is limited, and that attending to the adaptability and multiplicity of norms (Carel, 2007; Mol, 1998) can generate different appreciation of the social norms of exercise in ED recovery.

Moreover, it is important to incorporate social norms into discussions of exercise, as norms can be expressed not only through the material but also via the social. For example, exercising for permission to eat is a commonly cited symptom of dysfunctional exercise (Calogero & Pedrotty, 2004). However, a recent BBC horizon television program (*The Restaurant That Burns Off Calories*, which aired in April 2020) was based on the premise that every calorie eaten in the show’s restaurant had to be burned off afterward in the gym. This popular representation of exercise and dietary norms reinforces the idea that movement can or should be equated to food eaten or calories burned; attitudes that are very common among people with EDs. In this regard, many of the features of dysfunctional exercise are practiced and sanctioned in wider (particularly Western) cultural norms and values (Williams, 2012). Women in recovery from EDs are thus required to operate within a socio-political climate whereby disciplining the body through exercise is largely accepted as unproblematic. In addition, they are responsibilized for their own well-being through neoliberal logics that encourage a “DIY” approach to health and recovery (Rose, 2014).

Therefore, to understand dysfunction, we must look at not only the individual and their beliefs but also the cultural values that sustain these practices. As such, pathology (in Cangulheim’s terms) does not exist only in clinical constructions of EDs but also in the cultural fabric and social practices of contemporary life in which they are situated (Rose, 2009). In this article, we employ a phenomenological approach to understanding how these multiple cultural values are subjectively navigated by women attempting to steer a path through recovery.

## The Study

Female participants aged 17 and above and living in the United Kingdom, who had a history of EDs and were weightlifting during their recovery, were sought to take part in the study. A two-pronged approach to sampling was used to acquire a complete sample of *n* = 19. First, the study was advertised in a selected group of gyms in England. Recruitment through gyms and word of mouth yielded 10 participants. Second, calls for participants were posted on Instagram and Twitter and individuals who fitted the study criteria were identified and direct messaged through social media. Individuals were identified as potentially suitable to take part in the research through the searching of hashtags (e.g., “#edrecovery”) on Instagram, using an alias account created specifically for the study. The content author’s Instagram profile was then viewed, to search for further indications that would confirm the individual was using weightlifting as a mode of recovery from EDs. The most common evidence for this came in the form of text captions below photos, where the account holder explained their transition from an ED to weightlifting. On other occasions, the existence of the hashtags “#edrecovery” or “#recoverywarrior” beneath images of women in the gym or “flexing” in gym-wear was sufficient to justify initial contact. A total of nine participants were secured using this online recruitment method. Despite the perceived “hard to reach” nature of this population, recruitment was relatively easy—as the time from the start of recruitment to the end of the first round of interviews totaled 6 months.

The women who took part in this study were all amateur participants in a range of weight training styles. These included bodybuilding, strength training, powerlifting, strongwoman, and CrossFit. Participants engaged with one or a mixture of these activities on a weekly basis and had done so for a minimum of 8 months at the time of interview. In terms of ethnicity, 16 participants identified as White British, two as British Indian, and one as Chinese. Their ages range from 17 to 38 and they live in various locations across the United Kingdom.

In-depth interviews were conducted longitudinally; three with each participant, scheduled at 4-month intervals. Interviews were semi-structured and typically lasted between 45 minutes and 1.5 hours, with the exception of the third and final interview, which was more commonly a 20- to 30-minute conversation over phone/video call or email/messenger app. Interviews were scheduled longitudinally to capture the messy, often non-linear nature of recovery, which has been documented by numerous other scholars (D’Abundo & Chally, 2004; Eli, 2018; Lester, 2019; Musolino et al., 2020; Weaver et al., 2005). The nature of the interview questions varied at each timepoint. Generally, the first interview was designed to capture the participant’s history with ED, how they came about weightlifting, as well as their approach to recovery. While the second and third interviews sought to capture the phenomenon at hand in a more “everyday” sense, as well as any changes that might have been occurring in terms of recovery. All interviews were audio recorded and transcribed verbatim. To create an informal, conversational atmosphere, some personal experiences with exercise practices were shared by the interviewer when speaking with participants. These interactions served to redress the power imbalance inherent within the interviewer–interviewee dynamic, as well as develop rapport (Cotterill & Letherby, 1993).^1^ Women were asked to self-report their previous experience with EDs (with or without a clinical diagnosis) and recovery status. Nine participants reported to have struggled with anorexia nervosa, two with bulimia nervosa, two with binge ED, one with eating disorder not otherwise specified (EDNOS), and the remaining five suffered with a combination of the above. While a minority of participants considered themselves “recovered,” the majority self-identified as “in recovery,” despite often being weight-restored and therefore no longer considered clinically “at risk.” Two participants identified as “still suffering” with an ED and attempting to recover.

Just under half of the women who took part had never received any treatment or therapeutic support for their ED. Of the participants who had at one time accessed care, two were inpatients, three were outpatients, and the remainder saw a therapist or psychologist. However, many of these women reported struggling to engage with formal treatment, citing weightlifting instead as the key initiator of their recovery. Significantly, of the participants who had not received formal treatment, many reported to have tried to access support during their ED but were either turned away or put on waitlists lasting years. Several participants were told by health care professionals that they would not be referred to specialist care due to “not being thin enough.” Despite the range of experiences with treatment within the sample, there were no obvious differences in the valuing of exercise between women who had and had not received care for their ED. However, future research on this topic involving a purely clinical sample would be valuable.

Due to the stigmatized and potentially vulnerable nature of the research participants in this study, ethical considerations were embedded in the research design. Ethical approval was gained from the host institution and participants were provided with written informed consent before any data were collected. It was anticipated that discussing exercise and food during the interviews may be triggering or interfere with recovery. This was not found to be the case as, when asked to reflect on how the interview made them feel (immediately after the interview and in follow-up conversations), participants reported the process to be positive, as it allowed them space to reflect on their experiences in a non-therapeutic context. However, in situations where participants disclosed that they were struggling to recover, they were directed toward various resources such as ED helplines and information on how to access support.

### Analysis

We approached the data set using Interpretive Phenomenological Analysis (IPA), which asks the question “what is this kind of experience like?” (Shinebourne & Smith, 2009). This analytic approach is well suited to health-related research with small sample groups, as it seeks to interpret meaning from the depth and richness of personal narratives within a given phenomenon (Shinebourne & Smith, 2009). At its core, IPA is interested in experience, subjectivity, and relatedness, which are central to the research questions for this project. In this respect, a phenomenological approach is useful for providing an understanding of women’s agentic response to the systems of values in which they are situated.

To develop the three themes examined in the findings, we followed the three stages of IPA as described by Smith and colleagues (2009). The first stage of analysis involved “initial noting,” which involved writing down the first impressions of the data. During this process, the following questions acted as prompts; “what is being described?” “what kind of language is used?” and “what (at surface-level) is being communicated?” The second phase focused on accessing a deeper level of meaning by establishing patterns such as commonly used metaphors and the repetition of language. Here the specific terms used by participants to describe pathology or communicate understandings of health began to emerge. The third phase operated at the conceptual level whereby processes of meaning-making and overarching themes were identified.

### Structure and Managing Routines

Weightlifting punctuated participants’ daily lives and created a sense of routine from regular planned workouts. So, for example, participants knew that they would train legs on Monday, chest and back on Tuesday, squeeze out a cardio session “here and there,” and plan their rest days around their other responsibilities. Here, a participant explains the benefit of such a consistent approach:

It’s all about stability because as I mentioned, panic cardio and binging are all really inconsistent, infrequent, can happen at any time things, and that’s exactly what leads to yoyoing and binging . . . it’s that cycle that you can’t stop. But if you just do things consistently, it’s calming, it’s a routine, it becomes habitual and then you just feel safe. And you feel in a good place to not make yourself ill.

For the women in this study, “structure” was reassuring. This finding is supported by previous research which notes the significance of routine for women living with EDs and in recovery (Musolino et al., 2020; Weaver et al., 2005). For example, writing on women’s experiences with recovery, Weaver and colleagues (2005) contend,

staying on top in the real world is healthy eating amid the stress of everyday role responsibilities. It requires normalising eating (structuring daily intake to meet caloric requirements in such a way as to prevent starvation or bingeing) and self-monitoring (watching over intake and activity levels). (pp. 197–198)

The structured nature of weightlifting was associated with “calm” and “safety” and was used to compartmentalize exercise within the busyness of everyday life.

Participants were aware that engaging in too much exercise could lead to physical activity occupying too much space in their lives and, as a result, had learnt to adjust “obsessive” exercise. Somewhat paradoxically, this adjustment meant sticking to a consistent and sustainable routine which incorporated plenty of rest. Here, a participant speaks to how having a schedule helps them avoid slipping into obsessive territory:

I do get obsessive about things because I am quite determined and that can cross over into being obsessive and damaging. I think at the moment I counter it by . . . like my lifting programme is 4 days a week, so I literally have to take 3 days to chill and to not do anything . . . And I think training for performance helps that because the recovery side of things is just as important as the actual training. That stops it consuming everything because you’ve got to recover.

This participant’s desire for structure is effectively channeled into practices that are experienced as supportive of well-being. In the ED literature, however, such an emphasis on strict routine is typically pathologized (Calogero et al., 2010; Danielsen et al., 2018).

Significantly, the women in this study did not view their exercise routines as pathological, but as normal and as an integral to their sense of self. For example, participants self-identified as “type A,” “black and white” thinkers, and “perfectionist” in nature. These qualities (which have been well documented in the clinical literature; e.g., Wade et al., 2015) were conceived of as lifelong and therefore to be actively managed. As two participants noted,

It’s all about sort of distancing yourself from the obsession. Not making it an obsession but just making it a hobby—something that you enjoy. Because when you start getting obsessed, you don’t enjoy it anymore. You feel like you’re making yourself go [to the gym]. I do get like that sometimes.

You need to learn how to deal with your personality traits. Like, I’m always going to be a “type A” person but now I’ve found healthy ways of utilizing that, rather than turning to restriction instead.

In this respect, participants acknowledged the dangers associated with restrictive exercise practices yet asserted that these behaviors could be redirected toward well-being. Sholl (2015), writing on Canguilhem, suggests that “given the right conditions, [people are creative and are capable of] establishing a new norm, of adapting to changing demands” (p. 410). During recovery, the women in this study used the safety of structure and routine to re-inscribe exercise with positive values, thus establishing new embodied practices that served them (Wacker & Dolbin-MacNab, 2020).

### What the Body Can Do

A key finding from this research was that the subcultural norms within weightlifting that normalize and encourage eating and weight-gain were particularly beneficial to the recovery process. To improve within the sport of weightlifting, one is required to lift heavy weights on a routine basis, fuel the body with lots of food, and take regular rest to let the muscles of the body repair. For the women in this study, who were all amateur lifters, occupying a larger body translated into improvements in strength and performance, thus rendering weight-gain (in terms of both muscle and fat) legitimate within the context of their sport. In this respect, exercising to build strength and to take up space with their bodies was viewed by participants as supportive to recovery, as it so directly contradicted the restrictive and minimizing practices that sustained their EDs. As one participant described,

Author 1: What is it about weightlifting that has been useful for your recovery?

Participant: I think changing the mindset from wanting to lose weight to wanting to reach a goal. It does change your mindset to thinking like, I want to grow, I want to have more muscle. Rather than losing weight, it is about gaining weight and muscle.

In this context, participants’ weightlifting was driven by growth and progress which, within the subculture of the sport, are referred to as “gains.” Gains were experienced in two distinct senses of the word. First, there were “mental gains,” which speak to the satisfaction participants experienced by virtue of developing strength and reaching new athletic goals. Second, were “physical gains,” achieved through weight-gain and increased muscularity. These two tenets of growth, the mental and the physical, were entwined and mutually reinforcing. Typically, “mental gains” would occur thus normalizing and permitting the embodiment of “physical gains.” In this regard, a participant explained her reaction to the weight-gain that occurred when she first began weightlifting:

I think it was hard for a bit because just when I saw the difference in weight I was just like, oh my god, I’m going to have to lose all this extra weight I’ve put on. Then I was like wait a minute, how about I see how much I can lift now. And then when I built my deadlift up, I was like, woah I can deadlift way more!

Thus, within the “gains” framework, food and eating was rendered productive and positioned as working with the body, rather than against it. It was noted by participants that other sports (such as yoga, running, and dance) failed to provide this sense of confidence and self-acceptance in connection with occupying a larger body. For example, one participant (who identified as “still suffering” but attempting to recover) recounted that her therapist had recommended she practice yoga to aid her recovery. Here, she expresses her concerns:

You know, I’m a working-class second-generation immigrant, kid of refugees. Right? So I stick out like a sore thumb here where I live anyway. I grew up in Cambridge, which is completely white. But as an athlete, sport was my sort of . . . the thing that allowed me to fit in . . . But from a physical size and shape perspective, whereas in other classes I don’t care that I’m a bit bigger sometimes because I’m 10x stronger than most of the women and the men in there and that was enough for me. Whereas in yoga it kind of doesn’t matter [that I’m strong], so I find it harder . . .

For this population, engaging in exercise that provided permission to occupy a larger body was central to moving in ways that supported mental health. While yoga may “fit” as a sporting identity for some, for others, the culture surrounding yoga, which is highly feminized and perceived as White, middle-class and thin bodied, presents a barrier to engagement (Webb et al., 2017). Exercise, as Hardes (2018) argues, is not politically neutral, and it is deeply informed by class-based practices (Spotswood & Tapp, 2010). However, in scholarship that cites yoga as a beneficial exercise for women in recovery, yoga is decoupled from its cultural norms and values, which potentially alienate many women who do not feel at home in highly gendered, middle-classed environments.

Weightlifting is not exempt from gendered stereotypes, and being a female weightlifter presents its own challenges. Perhaps most saliently, muscles are viewed as masculine, which has created a culturally imposed “upper limit” to women’s size and strength. This is defined by Dworkin (2001) as the “glass-ceiling” of muscularity. Participants were aware of this “glass ceiling” and were resistant to the idea that there should be limits on women’s physicality:

I think there’s a lot of information to wade through as a girl whereas a boy it’s just like eat more, get bigger, bulk, get huge, do bench press, increase your size—it’s very much like a linear thing . . . and there are so many things like, people telling girls they should only do like low weight work and things like girls should just only do cardio . . . but for me, feeling physically stronger has made me a lot more confident.

Participants like the one quoted above came to reject certain values that were associated with their ED, such as restriction and shrinking their bodies down, and instead took up a vastly different set of values, premised on strength and taking up space.

It is noteworthy that this transition, from exercise performed with the purpose of attaining thinness to movement centered on strength and muscle-building, has been identified as a broader cultural trend by feminists and scholars of body image (Holland & Tiggemann, 2017), and is marked by a wealth of literature detailing the prevalence and measured effects of #thinspiration and #fitspiration content on social media (Griffiths et al., 2018). However, participants were generally ambivalent about the visual consequences of these transformations of the body. While exercising to achieve a certain “look” is highly normalized within Western fitness cultures (Hesse-Biber et al., 2006), the women in this study were tentative to attach too strongly to the aesthetic effects of weightlifting, as they viewed exercising for a certain image as a “slippery slope.” For example, one participant noted her own precarious relationship to body image and exercise:

I always like the idea of watching my body change in terms of its shape, in terms of its fat, in terms of its muscle, because that’s what I loved so much about it when I was poorly. But I think subconsciously, and probably consciously, I know that that’s probably a little dangerous for me, to do it for those reasons, specifically because I know I’ll probably run away with that and it could go down a bit of a slippery slope.

This participant’s hesitancy to fixate on her body’s image is reflected in ED literature, which problematizes exercising for appearance goals (Calogero et al., 2010). In this way, while eating and exercising have a direct impact on the body’s inherent mutability, women are required to disconnect from any visual transformations that take place, lest they become pathologized. The women in this study navigated this tension by focusing instead on functionality and changes in terms of what the body can do (Pereira Vargas & Winter, 2021). For example, one participant noted,

It’s a great sport and it’s really empowering—and it’s what kickstarted my recovery, being able to train for performance rather than aesthetics. You know, you can judge it in weights lifted I suppose, rather than my actual self . . . so that was a big thing and I just want to be a bit fitter at the minute . . . like, strong.

Participants channeled their desire for transformations of the self into satisfaction derived from “making and re-making their own bodies, their own bodily norms included, rather than [those] bodies and norms being nailed down in advance [by medicine]” (Philo, 2007, p. 89). However, in doing so, they also came into conflict with wider cultural norms that see fitness as a tool for weight-loss and body maintenance (Hesse-Biber et al., 2006). In this regard, women in recovery navigated multiple, contradictory norms; one for the medical establishment and one for wider culture (Mol, 1998).

### Regulating Affect

According to participants, “healthy” exercise was that which promoted embodied strength, happiness, and reduced stress. In this regard, discussions involving “endorphins,” “cleansing the mind,” and having “me time” were terms used to describe the beneficial effects of movement. Time spent weightlifting was often described as “mindless” and a space where “stressors in life melted away.” For example, one participant explained how weightlifting helped her clear her head of worries:

Author 1: Why do you use weightlifting to recover from your eating disorder?

Participant: Because I don’t think about my outside issues. As soon as I get in [the gym], nothing . . . is in my head—there’s no issues anymore. I literally am just there to do what I need to do . . . I don’t think about my deadlines for uni and I don’t think about my assignments or my ex-boyfriend or my eating. I just think about squats and deadlifts and that’s what I like.

Following a workout, participants reported to experience a more positive attitude as well as a sense of confidence, which is consistent with psycho-somatic research on the effects of exercise (Bernstein & McNally, 2018; Fox, 1999). While this may appear to be a constructive use of exercise for women in recovery, in the ED literature, exercising to regulate mood is identified as dysfunctional (Vansteelandt et al., 2007), due to the fact that affect regulation has been found to be an explanatory variable for “exercise dependence” in both ED patients and controls (Bratland-Sanda et al., 2011).

This framing of affect regulation as dysfunctional in ED groups is problematic. In the general population, regular exercise is heavily promoted due to being associated with fewer (and less severe) symptoms of depression and lower anxiety levels (Goodwin, 2003; Ströhle, 2009), and has been successfully used as an intervention among groups with various psychological issues (Schuch et al., 2016). Yet, in their writing on the relationship between affect regulation and exercise in the context of EDs, Vansteelandt and colleagues (2007) contend, “in our opinion, physical activity in eating disorders is not health promoting but pathological in nature” (p. 1728). In this regard, EDs are set apart from other forms of mental illness, due to the wholly pathological framing of exercise in this context.

Participants, however, did not experience weightlifting as illness-making, instead they intentionally used exercise to combat stress and feel happy. There was a coherent sense in which constructive exercise during recovery was that which fostered a greater sense of body awareness, as participants reported weightlifting helped them feel more attuned to physical sensations. For example, it was noted,

You feel your arms getting tired, in the past I would have probably disassociated from my body and would get a kind of numbness in my arms and legs, so I think part of it as well is kind of like, I am more aware of my body generally. But especially in the gym. I quite like that feeling the next day of having a little bit of an ache in my arms or a little bit of an ache in my abs . . . Yeah so I think it makes me more aware of myself and my body as well. And sort of that I am getting stronger, as my mind gets stronger.

When talking about exercise they experienced as healing and restorative, participants often provided affectual and embodied accounts, framed by heightened bodily sensations. One participant, for example, described her enjoyment of the feeling of soreness after a workout:

Author 1: What is it about that feeling that you enjoy?

Participant: It’s just my body saying, you targeted that area and you did a successful session and . . . like if I don’t feel sore, it’s still fine but then, when I feel a little bit achy it’s like, yeah I did well. I never push myself to the point of discomfort, I just go and I enjoy. But when I do feel like, oh I need to [foam] roll a little bit, it’s almost an achievement. Mm, not an achievement, because that feels quite disordered- when I think of pain as achievement . . . but it’s . . . I’m trying to say it in a way that doesn’t make it seem like I’m back with an eating disorder . . . um it’s just I know that I did well. And it’s my body saying . . . you know, we had a good session.

If we follow Cangulheim’s (1943/1966) focus on the appreciation of people’s realities and experiences, “pushing oneself” and feeling pain is not dysfunctional, but key to “the awareness of [one’s] body” (p. 46). As “the state of health is a state of unawareness” (Canguilhem, 1943/1966), attention to pain and bodily sensations produced by weightlifting brings awareness of health, and signals wellness (not sickness). In their writing on “healthy” exercise during ED recovery, Calogero and colleagues (2010) specifically advocate for movement that is performed with “attention, purpose, self-compassion, acceptance, awareness, and joy” (p. 434). It is noteworthy that missing from this list of values that designate the “correct” way of exercising are many of the normative principles associated with exercise culture generally, such as perseverance, “going for the burn,” controlled aggression, competition, “mind over matter,” and achievement.

It is significant that the construction of certain types of gendered exercise as having uniformly positive and “joyful” affect negates other expressive emotions and bodily movements. For women in this study, weightlifting supplied access to a sense of power, as well as providing a space to legitimately feel emotions such as frustration. As the ED literature suggests that emotional suppression and latent anger is linked to ED symptomology in women (Zaitsoff et al., 2002), our research demonstrates how exercise might be used as an enabling outlet for this kind of affectual experience. As one participant described,

Author 1: This might sound like a bit of an odd question, but how do you feel when you’re training?

Participant: I think it depends. If it’s going well, if it’s a good training day, I feel like “powerful” I guess and when I have my really loud music on and also I feel generally in the gym is when I’m my happiest . . . or I’m in a bad mood I can still feel really powerful doing it and it can be a way of getting out like frustration or if I’m training with friends it can be because I’m really happy and chilled. I just generally like the emotions associated with it.

Normative constructions of “healthy” exercise during eating recovery as being that which is joyful and centered on pleasure do much to obscure different yet equally legitimate forms of affect and embodied expression, such as anger and frustration, which may be more easily accessed through explosive, powerful, and strength-oriented movement. In this respect, yoga, which honors containment and the interiority of the self, may not offer the kind of affectual outlet women in recovery would benefit from.

## Conclusion

Female weightlifters in recovery from EDs face conflicting understandings of normal and pathological exercise behavior. In this article, we have demonstrated that navigating such tensions requires reconfiguring and mobilizing what in some settings is construed as pathological, yet for participants is transformed toward productive and supportive ends. In using the practice of weightlifting as a case study, we have demonstrated how a desire for structure and routine moves away from ED pathology toward normalization, as it is effectively leveraged by participants in service of recovery. Similarly, although attention to appearance and aesthetics is problematized in ED literature, women in recovery redirected this appetite for transformation toward watching their body grow in line with their functional ability and strength. Finally, while exercising to regulate affect is discouraged by clinicians, through weightlifting, participants found an outlet for latent anger, as well as opportunities to feel powerful.

The findings from this research may be of use to health practitioners in the ED field, as they have potential implications for treatment. In particular, this research speaks to the clinical understandings of “dysfunctional” exercise, and the tensions that arise when these definitions are positioned alongside wider cultural and social norms. It is worth considering that when exercise is experienced by women as restorative and supportive to the daily practice of recovery, this lived experience should surely displace or override pathological conceptions of that activity. In this respect, Canguilhem (1943/1966) writes,

is the concept of disease a concept of an objective reality accessible to quantitative scientific knowledge? Is the difference in value, which the living being establishes between his [*sic*] normal life and his [*sic*] pathological life, an illusory appearance which the scientist has the legitimate obligation to deny? If this annulling of a qualitative contrast is theoretically possible, it is clear that it is legitimate. (p. 76)

For Canguilhem, medical understandings of pathology should be responsive to adaption when positioned in the context of subjective experiences of health. As Mol (1998) argues, “engaging in medicine is a human activity” (p. 278) and there are a multiplicity of norms and values that are both subjectively ascribed and context dependent. Furthermore, as we have argued in this article, greater attention should be paid to how people adapt and respond to “conditions” in ways that create possibilities for value or joy (cf. Carel, 2007).

Finally, it is important to consider how normative gender ideals become embedded within medical knowledge and thus establish and fix understandings of “normal” behavior (Moulding, 2006). In this article, we have compared yoga, which is an activity imbued with highly feminized qualities, alongside weightlifting, which is a masculinized sport and more closely represents the norms within hegemonic physical culture. The advocation of yoga and relative dismissal of weightlifting speaks to gendered perceptions of the “correct” kind of movement to support women’s “emotional ailments” (Hardes, 2018, p. 191). In this respect, movement that is mindful, embodied, joyful, non-competitive, and social is preferred over exercise that requires commitment, is physically demanding, and offers an outlet for aggression. In fact, one could argue that, for women with EDs, it is precisely the latter that is needed, given the links drawn between EDs and restrictive gender roles (Bordo, 1993), emotional suppression (Zaitsoff et al., 2002), and feelings of powerlessness (Katzman, 1998).

While the empirical group studied in this research offers useful insights into the ways in which women with a history of EDs take a non-clinical approach to recovery, future research on this topic involving a fully clinical sample would be valuable. Furthermore, given that the central tension in this article is between clinical understandings of dysfunctional exercise and wider cultural exercise norms, it would be fruitful to compare the exercise behaviors and attitudes of clinically labeled “dysfunctional” female exercisers and women who engage in regular exercise without this pathological categorization. Such research could serve to further expose and interrogate overlaps between what is considered “normal” and “pathological” exercise, and the role of sociocultural elements in recovery, that we have highlighted here.

## Notes

1. The lead researcher conducted all interviews for this study due to having an “insider” status through her participation in amateur weightlifting. To create an informal, conversational atmosphere, some personal experiences with exercise practices were shared by the lead researcher when speaking with participants. This relevant experience was valuable when the technical details of weightlifting were discussed, as well as some of the embodied sensations. For example, Hockin-Boyers had knowledge of specific exercises and styles of training (e.g., “hypertrophy”), as well as personal experience with bodily sensations, such as delayed onset muscle soreness (DOMS), which helped develop a sense of shared understanding between interviewer and participant.
